# Progressive intertriginous plaques in a patient with fatigue and diarrhea

**DOI:** 10.1016/j.jdcr.2024.06.027

**Published:** 2024-07-23

**Authors:** Bahar Momin, Angela Martini, Jesse Fike

**Affiliations:** a Long School of Medicine, UT Health San Antonio, San Antonio, Texas; b Department of Dermatology, UT Health San Antonio, San Antonio, Texas

**Keywords:** candidal intertrigo, candidiasis, dermatology, dermatopathology, intertrigo, Langerhans cell histiocytosis, rash

## Case presentation

**Fig 1:**
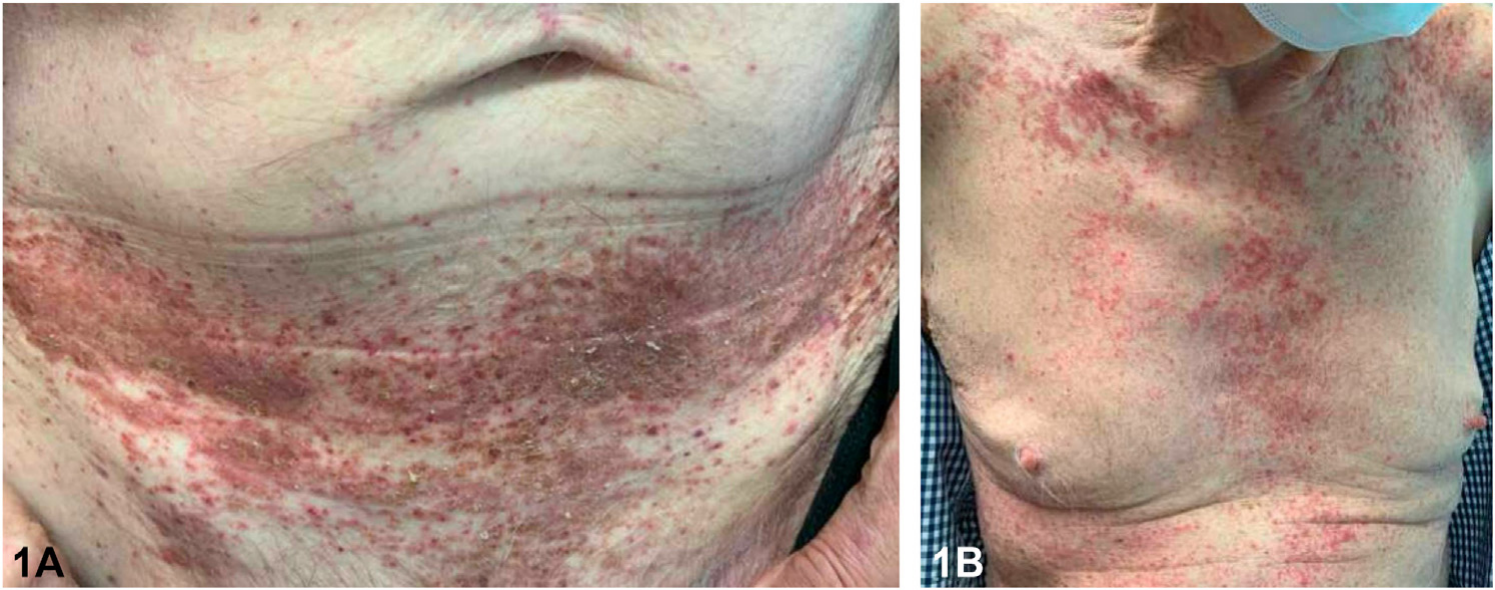

**Fig 2:**
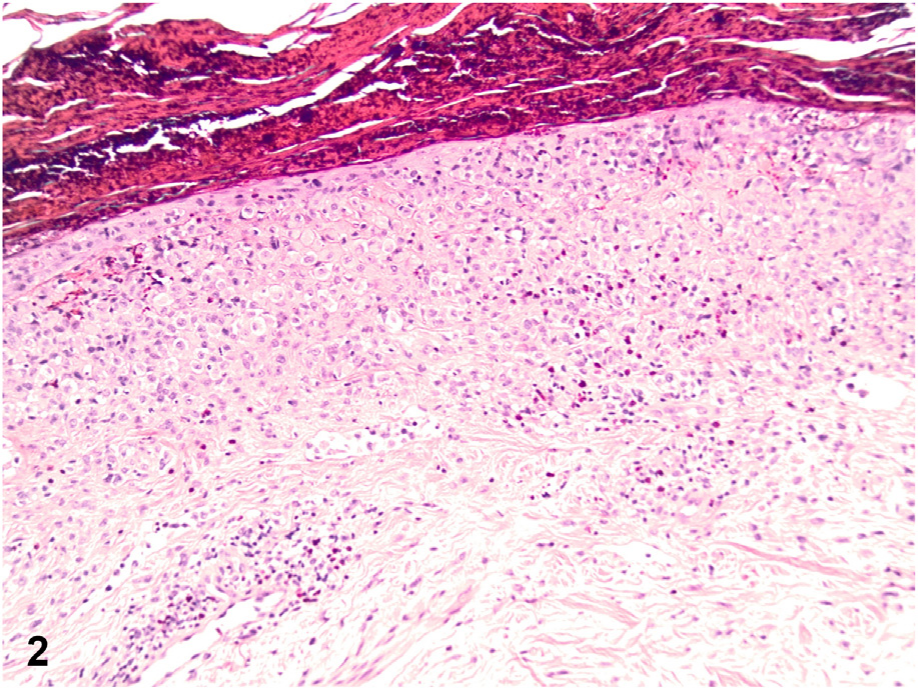

**Fig 3:**
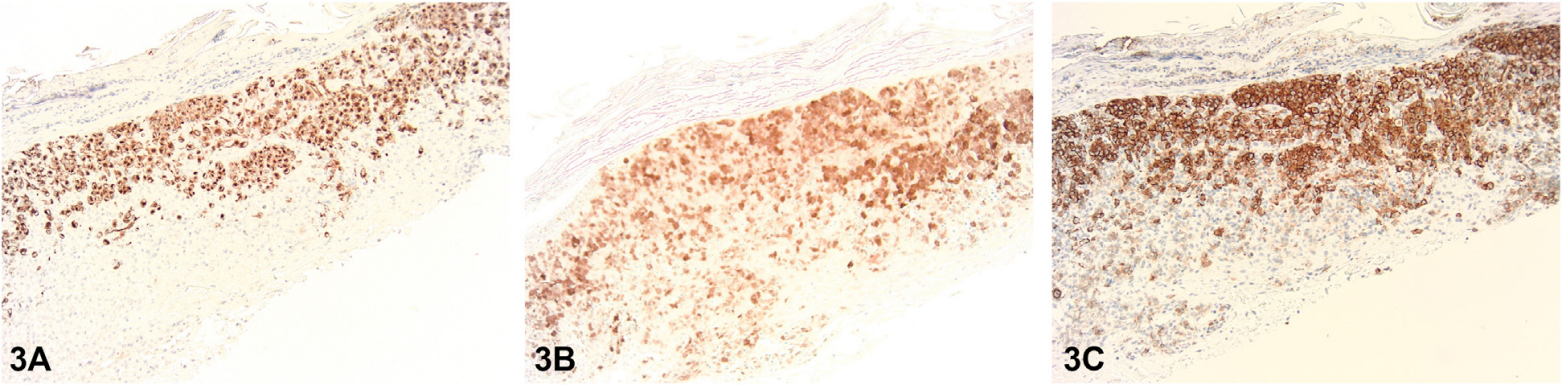

A 79-year-old male presented with a 1-year history of a pruritic rash to the intertriginous regions resistant to oral terbinafine. Initial examination showed erythematous plaques with satellite papules to the inguinal folds and suprapubic abdomen. Ketoconazole 2% cream, hydrocortisone 2.5% cream, and zinc oxide barrier ointment were started. Six-week follow-up demonstrated progression to eroded and crusted reddish-brown plaques throughout the groin, suprapubic abdomen, anterior trunk, upper mid-back, and neck (Fig 1). The patient also endorsed increasing fatigue and diarrhea. Shave biopsies demonstrated an intraepidermal collection of pale cells with reniform nuclei (Fig 2) positive for CD207, S100, and CD1a staining (Fig 3).

**Question 1: What is the most likely diagnosis?**

A. Acquired acrodermatitis enteropathica
B. Generalized eruptive histiocytosis
C. Langerhans cell histiocytosis (LCH)
D. Indeterminate cell histiocytosis
E. Multicentric reticulohistiocytosis

**Answers:**

A. Acquired acrodermatitis enteropathica – Incorrect. Acquired acrodermatitis enteropathica, a result of zinc deficiency, classically presents with an erosive intertriginous and acral eruption, diarrhea, and depression. Histology shows upper epidermal pallor and negative staining for CD207, S100, and CD1a.
B. Generalized eruptive histiocytosis – Incorrect. While generalized eruptive histiocytosis can present with widespread red-brown papules in an axial distribution, it stains negatively for S100, CD1a, and CD207 as it is a non-LCH.
C. Langerhans cell histiocytosis (LCH) – Correct. LCH, a rare clinically heterogeneous group of disorders, involves the proliferation of Langerhans cells within one or multiple organs. Primarily presenting in children, it can manifest at any age and in any organ. Cutaneous involvement classically presents with red-brown papules in the seborrheic distribution that become crusted, eroded, and/or petechial. Systemic involvement can be asymptomatic or exhibit constitutional symptoms and/or signs of organ dysfunction. Diagnosis requires clinical correlation with histopathology and immunohistochemistry. Histologically, a dense population of Langerhans cells with classic reniform nuclei can be seen within the papillary dermis. Immunohistochemistry stains are positive for S100, CD1a, and CD207.
D. Indeterminate cell histiocytosis – Incorrect. Indeterminate cell histiocytosis can present with generalized red-brown papules and stains positively for S100 and CD1a like LCH, however negatively stains for CD207.
E. Multicentric reticulohistiocytosis – Incorrect. Multicentric reticulohistiocytosis classically presents with acral and periungual red-brown nodules, destructive arthritis, and an increased risk of solid organ malignancy. Histology shows histiocytes with pink-purple ground glass cytoplasm, often surrounded by lacunae. It stains negatively for S100, CD1a, and CD207.

**Question 2: What mutation is most often associated with LCH?**

A. G protein subunit alpha Q/ G protein subunit alpha 11
B. C-kit
C. Phosphatase and tensin homolog (PTEN)
D. Rearranged during transfection
E. V-raf murine sarcoma viral oncogene homolog B1 (BRAF)

**Answers:**

A. G protein subunit alpha Q/G protein subunit alpha 11 – Incorrect. G protein subunit alpha Q/G protein subunit alpha 11 mutations are associated with uveal melanomas, nevus of Ota, and blue nevi, including malignant blue nevi with concomitant BRCA-1 associated protein 1 loss. These mutations have also been associated with port-wine stains, dermal melanocytosis, phakomatosis pigmentovascularis, and congenital hemangiomas.
B. C-kit – Incorrect. C-kit mutations have been associated with piebaldism, acral and mucosal melanomas, mastocytosis, gastrointestinal stromal tumors, germ cell tumors, and hematopoietic neoplasms such as acute myeloid leukemia.
C. PTEN – Incorrect. Mutations in the PTEN tumor suppressor gene have been associated with Cowden syndrome, Bannayan-Riley-Ruvalcaba syndrome, PTEN-related Proteus syndrome, and Proteus-like syndrome.
D. Rearranged during transfection – Incorrect. Mutations in the rearranged during transfection proto-oncogene have been associated with forms of multiple endocrine neoplasia type 2 and an increased risk of medullary thyroid carcinomas.
E. BRAF – Correct. Immunostaining was positive for BRAF V600 E mutation in our patient. LCH is characterized by a clonal proliferation of myeloid dendritic cells due to possible constitutive activation of the BRAF-driven mitogen-activated protein kinases signaling pathway. BRAF V600 E mutation has been reported in up to 57% of cases of LCH.^1^ There are emerging data on the use of BRAF and mitogen-activated protein kinase kinase 1 inhibitors in LCH cases with proven BRAF mutations, with the majority having a complete remission throughout the duration of therapy.^2^ BRAF V600 E mutations are also implicated in approximately 50% of cutaneous melanomas.^3^

**Question 3: What is the most appropriate next step in diagnostic workup?**

A. Imaging
B. Electron microscopy
C. Systemic corticosteroids
D. Radiation
E. None of the above

**Answers:**

A. Imaging – Correct. After histopathologic confirmation of LCH, evaluating for systemic involvement is essential as 70% of adults with LCH have multisystem involvement.^4^ It has been highly associated with hematologic and solid malignancies with a frequency of up to 32% in adults.^5^ In our patient, computed tomography imaging suggested possible pulmonary, splenic, small lymph node, and bowel involvement. However, bone marrow biopsy showed no evidence of LCH. The patient was placed on enzyme supplementation after diagnosis of exocrine pancreatic insufficiency possibly secondary to LCH.
B. Electron microscopy – Incorrect. Although identification of cytoplasmic Birkbeck granules on electron microscopy may support the diagnosis of LCH, it is not required.
C. Systemic corticosteroids – Incorrect. Treatment of LCH varies based upon single versus multiple organ system involvement, which is why evaluating for multisystem involvement is the next important step. Localized skin involvement may be treated topically with steroids and, in refractory cases, there has been some reported success with photochemotherapy, narrowband ultraviolet B therapy, imiquimod, and nitrogen mustard.^2^ More extensive skin involvement often responds to methotrexate or hydroxyurea, which are also treatment options for the multisystem disease.^2^
D. Radiation – Incorrect. Radiation can be utilized for LCH with bone involvement, which was not seen in our patient.
E. None of the above – Incorrect. In adults, multisystem involvement of LCH is common with organ (liver, spleen, hematopoietic) involvement being associated with increased mortality of up to 20%.^5^ Therefore, thorough evaluation is vital in the management of the disease.

## Conflicts of interest

None disclosed.
